# Determination of Slope Safety Factor with Analytical Solution and Searching Critical Slip Surface with Genetic-Traversal Random Method

**DOI:** 10.1155/2014/950531

**Published:** 2014-03-17

**Authors:** Wen-jie Niu

**Affiliations:** College of Mechanics and Engineering Department, Liaoning Technical University, Fuxin, Liaoning 123000, China

## Abstract

In the current practice, to determine the safety factor of a slope with two-dimensional circular potential failure surface, one of the searching methods for the critical slip surface is Genetic Algorithm (GA), while the method to calculate the slope safety factor is Fellenius' slices method. However GA needs to be validated with more numeric tests, while Fellenius' slices method is just an approximate method like finite element method. This paper proposed a new method to determine the minimum slope safety factor which is the determination of slope safety factor with analytical solution and searching critical slip surface with Genetic-Traversal Random Method. The analytical solution is more accurate than Fellenius' slices method. The Genetic-Traversal Random Method uses random pick to utilize mutation. A computer automatic search program is developed for the Genetic-Traversal Random Method. After comparison with other methods like slope/w software, results indicate that the Genetic-Traversal Random Search Method can give very low safety factor which is about half of the other methods. However the obtained minimum safety factor with Genetic-Traversal Random Search Method is very close to the lower bound solutions of slope safety factor given by the Ansys software.

## 1. Introduction

The geotechnical engineer frequently uses limit equilibrium methods of analysis when studying slope stability problems, for example, Ordinary or Fellenius' method (sometimes referred to as the Swedish circle method or the conventional method), Simplified Bishop method, Spencer's method, Janbu's simplified method, Janbu's rigorous method, Morgenstern-Price method, or unified solution scheme [1–3]. In order to reduce the influence of the assumptions made in limit equilibrium methods on the factor of safety, the methods of limit analysis based on the rigid body plasticity theory were developed by Chen (1975), Michalowski (1995), and Donald and Chen (1997). These methods based on the upper bound theorem of limit analysis are generally referred to as the upper bound methods, which give an upper bound solution to the real value of the factor of safety [4–6].

In the current practice, searching methods for the critical slip surface is a central issue to slope stability analysis. Previous research employed the Variational Calculus, the dynamic programming, alternating variable methods, the Monte Carlo technique, or the genetic algorithm (GA) into slope stability analysis for critical surface identification [7–13].

In recent years, genetic algorithm search procedure has been used to locate the critical slip surface of homogeneous slopes. It has been found that genetic algorithm is a robust search technique which often gives global solution [14, 15]. Numerical example shows that analyzing method of the slope stability based on the genetic algorithm is a global optimal procedure that can overcome the drawbacks of local optimum widely existing in classical searching methods and the result is satisfactory [16]. Established slope stability analysis methods cope well with moderately noncircular shear surfaces, and the simple genetic algorithm (SGA) has been used successfully to find the critical slip surface [17].

In the GA, the parameters in the optimization problem are translated into chromosomes with a data string (binary or real). A range of possible solutions is obtained from the variable space and the fitness of these solutions is compared with some predetermined criteria. If a solution is not obtained, a new population is created from the original (parent) chromosomes. This is achieved using “crossover” and “mutation” operations. Crossover involves gene exchange from two random (parent) solutions to form a child (new solution). Mutation involves the random switching of a single variable in a chromosome and is used to maintain population diversity, as the process converges towards a solution [18].

GA includes inheritance, mutation, selection, and crossover [19]. One of the core techniques and advantages of GA is that mutation can consider a wide range of possible solutions if natural evolution continues and never ends. The other advantage is that inheritance and crossover can save all the examples and virtues of the past age and pass them into the next generation to save time for the best choice [18, 19].

However there are many unsolved problems about GA in slope stability analysis. For example, how to realize GA with hand calculation method? How to realize GA with automatic search program? What is the relationship between GA and traversal random search method? How to improve the Fellenius method of slices concerning that the slices method is an approximate method like finite element method? In fact, all these problems are mathematical problems to find the minimum safety factor of a slope. These mathematical problems originated from the physical equations representing the common law of nature in slopes (e.g., Mohr-Column criterion of soil, 2D circular slip surface of homogeneous clay slope, and safety factor definition which is the moment of sliding resistance divided by the moment of sliding force). The physical laws of nature can be found and validated with repeated in situ or lab experiments to measure the physical quantities and mathematical logic to reveal the relationship. However mathematical problems can only be solved with logic deduction and validated with countless numeric tests.

This paper first intends to determine a cohesive soil slope safety factor with Fellenius' slices method, while the 2D critical failure surface is searched with GA. The analysis uses real-coded methods to encode the chromosomes with the variables of potential critical surface locations. The fitness of each chromosome is determined using the objective function that the resulting safety factors should be lower enough, and the fitness of all solutions is compared, while the chromosome of large safety factors shall be deleted [18]. However this part is realized with hand calculation.

Then a computer automatic search program (Genetic-Traversal Random Search Method) inspired by GA is made. The Genetic-Traversal Random Search Method presented in this paper only utilizes the mutation and selection thought of the traditional genetic algorithm. Crossover is omitted due to the difficulty in computer program realization and compensated with numerous random candidates due to mutation. The Genetic-Traversal Random Search Method makes a traversal search with random method. In the program, random numbers for random search are generated by computer and search boundaries are included. In the program, each slope safety factor is given by analytical solution rather than slices method. The safety factor and failure circle determination program developed in Silverfrost FTN95 is presented in the Appendix. At last, the proposed Genetic-Traversal Random Search Method is compared with other solutions such as slope/w software.

## 2. A Slope Stability Problem Example

A cohesive soil slope with its height 25 meters has a slope ratio of 1 : 2. The soil unit weight *γ* is 20 KN/m^3^. The soil internal friction angle *φ* is 26.6 degrees, and cohesion is 10 KPA. The problem now is to give the safety factor of the slope with a 2D circular failure surface.

## 3. Search the Critical Slip Surface with GA Method While Determining the Safety Factor with Fellenius' Method of Slices with Hand Calculation

To solve the engineering problem in Section 2, this part will search the critical slip surface with GA Method while determining the safety factor with Fellenius' method of slices.

### 3.1. The Slope Safety Factor with Fellenius' Method of Slices

**Figure 1:**
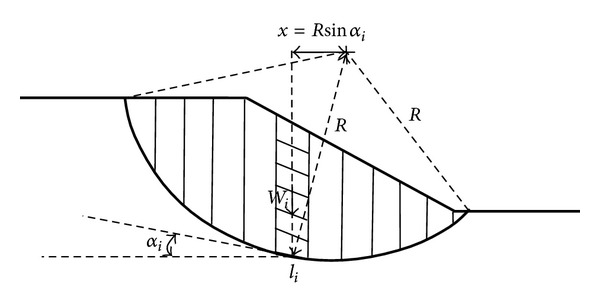
Fellenius' method of slices.

The potential slip surface for clay slope is two dimensional and a part of circle. In order to determine the slope safety factor in Figure 1, Fellenius' method of slices divides the slope into several slices [3]. Using moment equilibrium, the slope safety factor SF in Figure 1 is

(1) $$\begin{array}{c} \text{SF}=\frac{\sum \left(c_{i}+W_{i}\cos\alpha_{i}\tan\varphi_{i}\right)l_{i}}{W_{i}\sin\alpha_{i}}, \end{array}$$

where *c*_*i*_ and *φ*_*i*_ are the soil slope slice cohesion and internal friction angle. *W*_*i*_ is the soil slope slice self-gravity. *l*_*i*_ is the soil slope slice slip circular arc length. *α*_*i*_ is the angle between soil slope slice slip surface tangent line and the horizontal line.

### 3.2. Searching the Critical Failure Surface with GA

**Figure 2:**
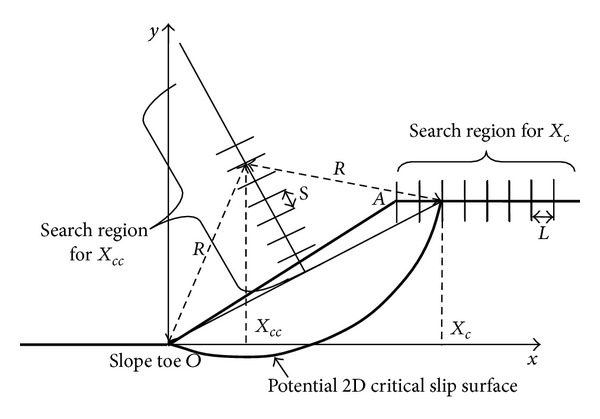
2D potential failure surfaces.

The assumed 2D slope failure surface is circular determined by two variables *X*_*c*_ and *X*_*cc*_ in Figure 2. *X*_*c*_ is the abscissa of a point on slope top surface. If *X*_*c*_ is determined, the 2D critical slip surface circle center with abscissa *X*_*cc*_ must lie on the perpendicular bisector of the straight line from the point of *X*_*c*_ to the slope toe *O*. *X*_*cc*_ is the abscissa of the critical slip surface circle center. So if *X*_*cc*_ is given, then the critical slip surface circle center can be determined. Altogether, if *X*_*c*_ and *X*_*cc*_ are given, 2D circular slope slip surface is determined.

However, in the next section, all the computations according to GA are made by hand calculations. If, for computer simulation, “interval" is as *L* and *s* as in Figure 2 and random numbers of computer function can help for automatic generation of variables *X*_*c*_ and *X*_*cc*_ in Figure 2, search regions or boundaries for *X*_*c*_ and *X*_*cc*_ must be defined with the definition of the limits of *X*_*c*_ and *X*_*cc*_. The necessity of these boundaries is evident because computer program must avoid generating surfaces out of the region of primary interest [21].

The searching process for the 2D critical failure surface as in Figure 1 uses techniques inspired by natural evolution, such as inheritance, mutation, selection, and crossover [19]. In a genetic algorithm, a population of strings (called chromosomes or the genotype of the genome), which encode candidate solutions (called individuals, creatures, or phenotypes) to an optimization problem, is evolved toward better solutions [19]. In this 2D critical failure surface searching problem, the candidate solutions are represented as {*X*_*c*_, *X*_*cc*_} described before. The evolution starts from a population of randomly generated individuals as {*X*_*c*_, *X*_*cc*_} and happens in generations. In each generation, the fitness of every individual in the population is evaluated; multiple individuals are stochastically selected from the current population (based on their fitness) and modified (recombined and possibly randomly mutated) to form a new population. The evaluation standard is that the individual {*X*_*c*_, *X*_*cc*_} with large safety factor is deleted and the individual {*X*_*c*_, *X*_*cc*_} with small safety factor is reserved. The new population is then used in the next iteration of the algorithm [19]. The algorithm terminates when a satisfactory fitness level has been reached for the population which means that it is hard to lower safety factor with iterations.

### 3.3. Searching Process with GA

In the GA, with hand calculation method, the potential failure surfaces for search are restricted that they all pass slope toe as in Figure 2 for simplifying the search task. Search process for the critical slip surface with genetic algorithm was presented from Table 1 to Table 12. In these tables, the minimum safety factors are marked with ∗ in each iteration. The units for *X*_*c*_ and *X*_*cc*_ are meters. Evaluation of individuals in Table 1 now begins. The evaluation standard is that the individual {*X*_*c*_, *X*_*cc*_} with large safety factor is deleted and the individual {*X*_*c*_, *X*_*cc*_} with small safety factor is reserved. Selection result will be put in Table 2. Crossover of selected individuals of Table 2 will be put in Table 3. Mutation begins and results will be put in Table 4. Evaluation of all previous individuals marked with ∗ begins. The evaluation standard is that the individual {*X*_*c*_, *X*_*cc*_} with large safety factor is deleted and the individual {*X*_*c*_, *X*_*cc*_} with small safety factor is reserved. Selection result will be put in Table 5. Crossover of selected individuals of Table 5 begins. Crossover result will be put in Table 6. Mutation begins and the result will be put in Table 7. Evaluation of all previous individuals marked with ∗ begins. The evaluation standard is that the individual {*X*_*c*_, *X*_*cc*_} with large safety factor is deleted and the individual {*X*_*c*_, *X*_*cc*_} with small safety factor is reserved. Selection result will be put in Table 8. Crossover of selected individuals of Table 8 begins. Crossover result will be put in Table 9. Mutation begins and result will be put in Table 10. Evaluation of all previous individuals marked with ∗ begins. The evaluation standard is that the individual {*X*_*c*_, *X*_*cc*_} with large safety factor is deleted and the individual {*X*_*c*_, *X*_*cc*_} with small safety factor is reserved. Selection result will be put in Table 11. The GA procedure terminates when a satisfactory fitness level has been reached for the population which means that it is hard to lower safety factor with iterations. The final result is in Table 12.

### 3.4. Location of the Critical Failure Surface and Safety Factor with GA Procedure

The example was solved with foregoing GA procedure. The minimum safety factor was 1.325 with *X*_*c*_ = 53 m and *X*_*cc*_ = 0. The corresponding slip circle center is at (0, 68.8 m) and the radius is 68.8 m.

## 4. Search the Critical Slip Surface with Genetic-Traversal Random Search Method While Determining the Safety Factor with Analytical Method 

To solve the engineering problem in Section 2, this part will search the critical failure surface with Genetic-Traversal Random Search Method while determining the safety factor with analytical method. This part is realized with computer automatic search program.

### 4.1. Analytical Method to Determine the Slope Safety Factor in the Above-Mentioned Slope Example in Section 2 [22]

**Figure 3:**
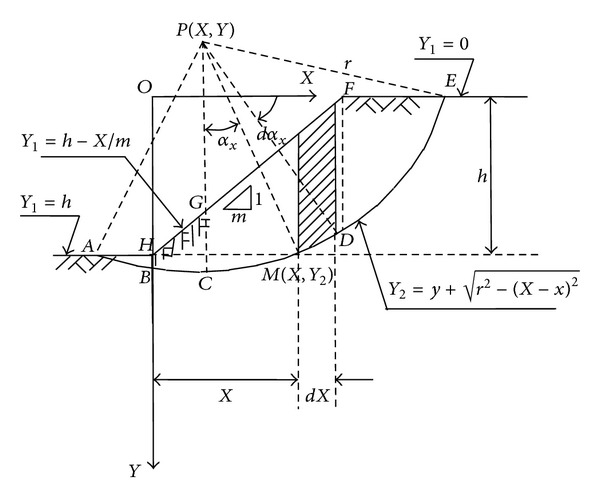
Analytical method to determine the slope safety factor.

With Fellenius' method, according to Zhang (1987), the analytical solution to give the safety factor in Figure 3 is

(2) $$\begin{array}{c} k=\frac{\gamma \cdot tg\varphi \left[N\right]+c\left[L\right]}{\gamma \left[T\right]}, \end{array}$$

where [*N*], [*L*], and [*T*] were given as

(3) N={[4r2−y2]r2−y2+[4r2−(h−y)2]×r2−(h−y)2+1m(2r2+x2)r2−x2−1m[2r2+(mh−x)2]r2−(mh−x)2}+r2{yarcsinr2−y2r−(h−y)arcsinr2−(h−y)2r+xmarcsinxr−mh−xmarcsinmh−xr},T=16r[3hr2−y3−(h−y)3−x3m−(mh−x)3m],L=r[arcsinr2−y2r+arcsinr2−(h−y)2r],

where, in Figure 3, *P*(*x*, *y*) is the potential failure circle center, *r* is the circle radius, *m* is the slope ratio, *h* is the slope height, *γ* is the slope soil unit weight, *φ* is the soil internal friction angle, and *c* is slope soil cohesion.

### 4.2. Genetic-Traversal Random Search Method

**Figure 4:**
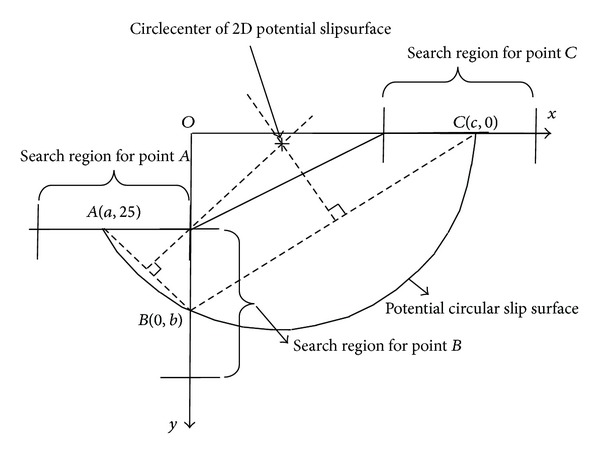
Potential failure circle center and radius determined and represented with points *A*, *B*, and *C*.

The slope stability problem example in Figure 4 is just the engineering problem in Section 2. Inspired by the genetic algorithm, the potential failure circle is represented with points *A*, *B*, and *C* in Figure 4. The coordinates of *A*, *B*, and *C* are (*a*, 25), (0, *b*), and (*c*, 0), respectively. So, in fact, the parameters *a*, *b*, and *c* can represent the potential failure circle. In a novel Fortran program, points *A*, *B*, and *C* are varied randomly and helped with random number generator subprogram. However, points *A*, *B*, and *C* can only vary in a certain region with boundary. Each group of {*a*, *b*, *c*} gives a safety factor by (2). With random number generator subprogram and loop program, enough groups of {*a*, *b*, *c*} are generated. Inspired by the genetic algorithm, the relative low safety factor and corresponding {*a*, *b*, *c*} are saved after each comparison between the old potential failure circle and the new generated potential failure circle and helped with the random number generator subprogram. After enough times of iterations set by the user, the minimum safety factor and corresponding {*a*, *b*, *c*} will be determined.

The safety factor and failure circle determination program developed in Silverfrost FTN95 was presented in the Appendix. In fact, the computer-aided genetic algorithm of the program presented in the Appendix only utilizes the mutation and selection thought of the traditional genetic algorithm. Crossover is omitted due to the difficulty in computer program realization and compensated with numerous random candidates due to mutation. In fact, genetic algorithm (GA) is a random search method based on the biological evolution law.

### 4.3. Results of the Program in the Appendix according to Genetic-Traversal Random Search Method for the Above-Mentioned Slope Stability Problem Example

After 100,000 times of potential failure circles' generation and selection, the obtained minimum safety factor is 0.648280, and the corresponding {*a*, *b*, *c*} is {−11.8283, 32.7429, 50.4410}.

## 5. Compared with Other Solutions

In order to validate the analytical solution to give safety factor of a specified slip surface, Genetic-Traversal Random Search Method to search for the critical failure surface and the corresponding program presented in the Appendix, this part will solve the slope engineering problem in Section 2 with other methods.

### 5.1. Solution of Searching the Critical Slip Surface with Fellenius' Method While Determining the Safety Factor with Fellenius' Method of Slices

To solve the engineering problem in Section 2, this part will search the critical failure surface with Fellenius' method while determining each safety factor with Fellenius' method of slices.

In this solution, safety factor is determined with Fellenius' method of slices as in (1). Fellenius' method of searching for the critical failure surface [20] was given as follows.

**Figure 5:**
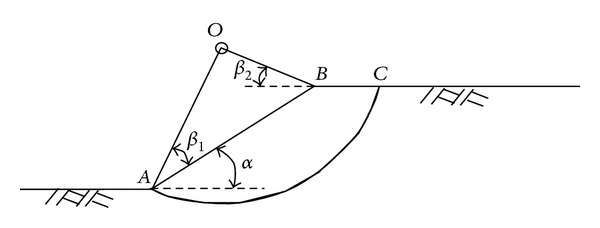
Determination of 2D potential failure surfaces when internal friction angle *φ* = 0.

If soil internal friction angle *φ* = 0, 2D critical failure surface passes through slope toe *A* and can be determined by Figure 5 and Table 13. In Figure 5, the critical failure surface circle center *O* can be determined by angles *β*_1_ and *β*_2_ which can be determined by slope angle *α* as in Table 13. Angle *β*_1_ is the angle between line *AO* and slope surface line, while *β*_2_ is the angle between line *OB* and slope horizontal top surface line *BC*. Point *B* is the intersection between slope surface line *AB* and slope horizontal top surface line *BC*.

**Figure 6:**
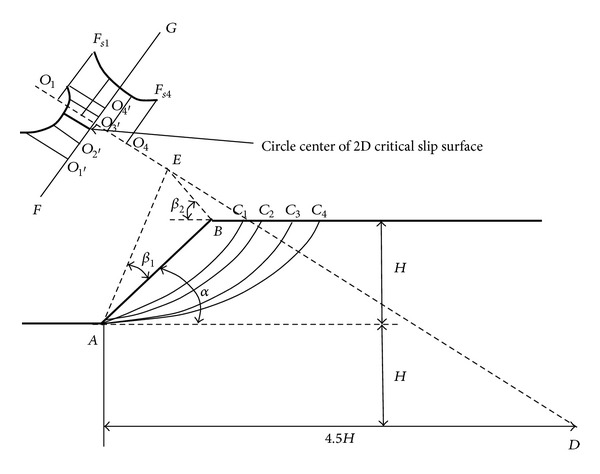
Determination of 2D potential failure surfaces when internal friction angle *φ* > 0 [20].

If soil internal friction angle *φ* > 0, 2D critical failure surface passes through slope toe and can be determined by Figure 6. In Figure 6, point *E* is determined by angles *β*_1_ and *β*_2_ which can be determined by slope angle *α* as in Table 13. The critical failure surface circle center may be on the extension line of the line *DE*. You can try many points on the line *DE* as the critical failure surface circle center candidate like *O*_1_ and *O*_4_ on the line *DE*. If a point *Ox* on the line *DE* is found to be the point which gives the minimum slope safety factor, then draw a line *FG* perpendicular to the line *DE* through the point *O*_*x*_. Then you can try many points on the line *FG* as the critical failure surface circle center candidate like *O*_1_′, *O*_2_′, *O*_3_′, and *O*_4_′. If a point on the line *FG* gives the minimum slope safety factor, this point means the one that gives the final most minimum safety factor of the studied slope.

The determined minimum safety factor with Fellenius' method is 1.320, while the 2D critical failure surface circle center is 4.5 m, 57.776 m, in the *x*-*y* coordinate system of Figure 2 and the radius is 57.951 m. The corresponding *X*_*c*_ in Figure 2 is 52.292 m.

### 5.2. Solution with Slope/w Software

To solve the engineering problem in Section 2, this part will determine the safety factor with slope/w software.

**Figure 7:**
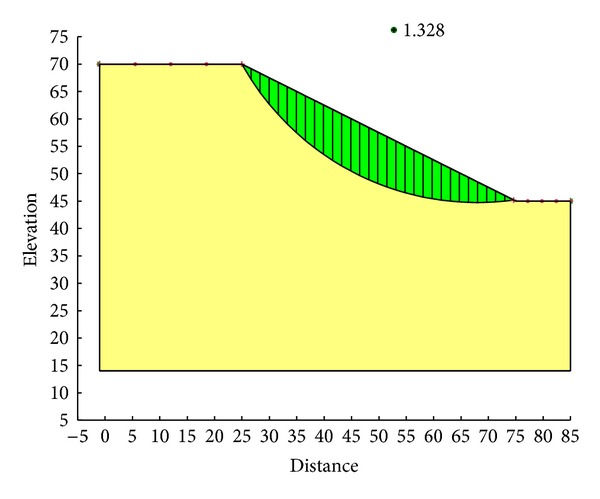
Critical failure surface with the Ordinary method.

**Figure 8:**
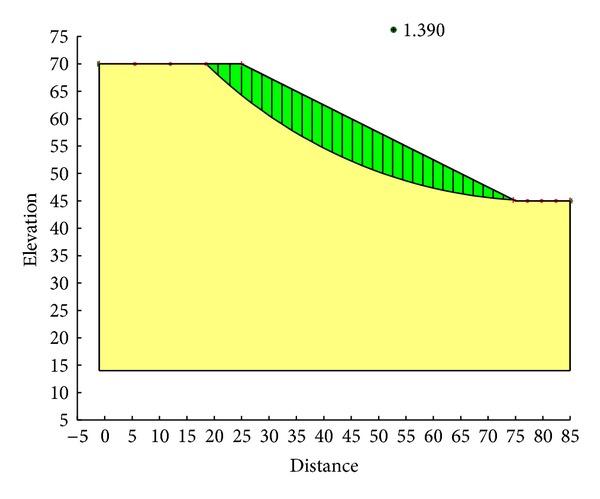
Critical failure surface with the Bishop method.

**Figure 9:**
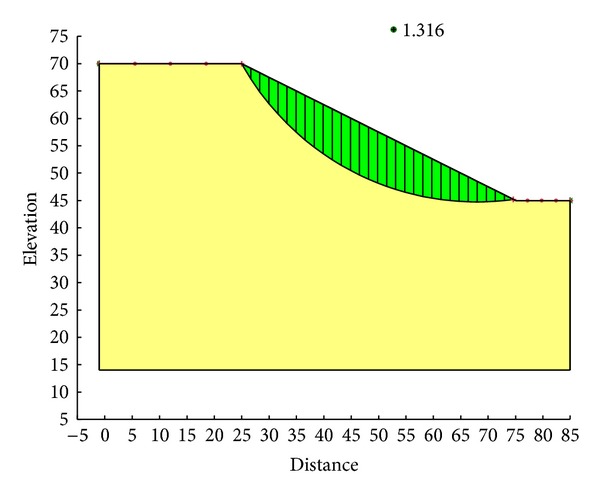
Critical failure surface with the Janbu method.

**Figure 10:**
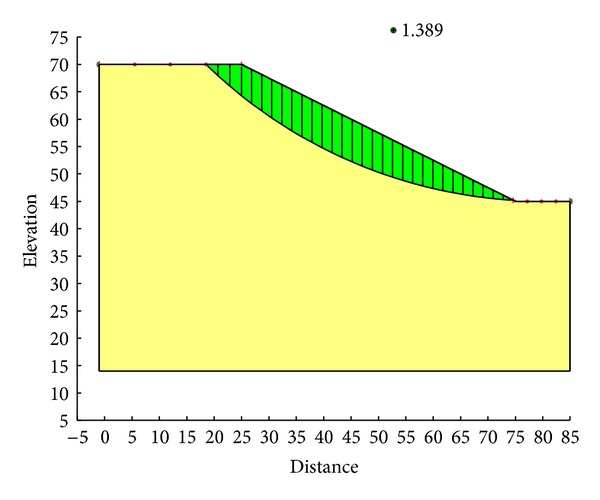
Critical failure surface with the Morgenstern-Price method.

**Figure 11:**
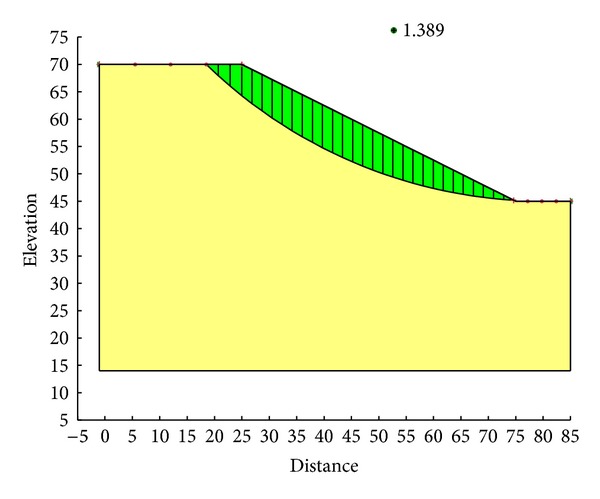
Critical failure surface with the Spencer method.

**Figure 12:**
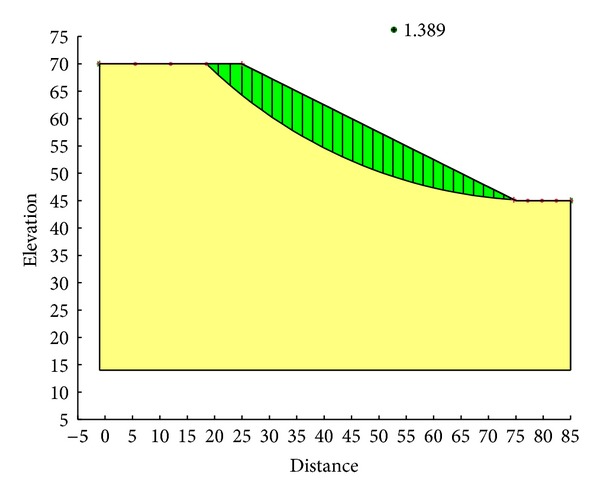
Critical failure surface determined with the GLE method.

**Figure 13:**
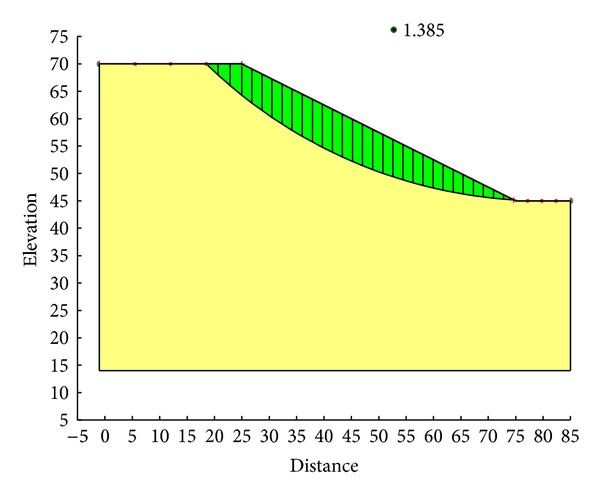
Critical failure surface determined with the Janbu generalized method.

With the Ordinary method, the minimum safety factor to the example slope is 1.328. The corresponding critical failure surface is presented in Figure 7. With the Bishop method, the minimum safety factor to the example slope is 1.390. The corresponding critical failure surface is presented in Figure 8. With the Janbu method, the minimum safety factor to the example slope is 1.316. The corresponding critical failure surface is presented in Figure 9. With the Morgenstern-Price method, the minimum safety factor to the example slope is 1.389. The corresponding critical failure surface is presented in Figure 10. With the Spencer method, the minimum safety factor to the example slope is 1.389. The corresponding critical failure surface is presented in Figure 11. With the GLE method, the minimum safety factor to the example slope is 1.389. The corresponding critical failure surface is presented in Figure 12. With the Janbu generalized method, after solving and analyzing, then after selecting the critical slip surface with a safety factor of 1.389 in the slope/w software, Figure 13 appears and the “minimum factor of safety” shows that its value is 1.385. 1.385 is not identical with 1.389 which is a little weird. The minimum safety factor determined by foregoing GA procedure is 1.325. After compared with slope/w software, foregoing GA procedure employed to search the critical failure surface is reasonable and applicable.

### 5.3. Solution with Ansys Software

**Figure 14:**
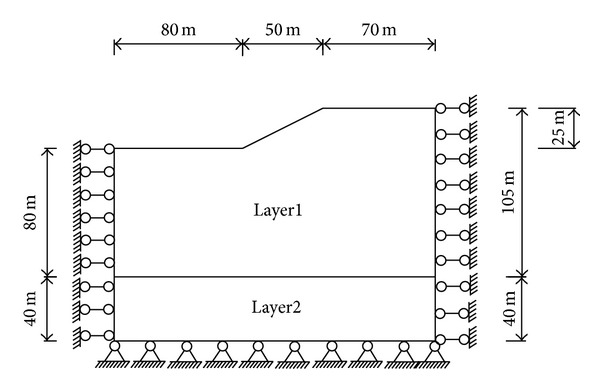
Studied region for the engineering problem in Section 2 treated with Ansys.

To solve the engineering problem in Section 2, this part will determine the safety factor with Ansys software [23]. The slope has two layers which is layer 1 and layer 2 in Figure 14. Layer 1 is clay and Layer 2 is bed rock. The slope layer 1's soil modulus of elasticity is assumed to be 2.0E7 N/m^2^. The slope layer 1's soil Poisson's ratio is assumed to be 0.3. The slope layer 1's soil density is assumed to be 2040.8 Kg/m^3^. The slope layer 1's soil cohesion is 10000 Pa and friction angle is 26.6 degrees. The slope layer 2's soil modulus of elasticity is assumed to be 3.2E10 N/m^2^. The slope layer 2's soil Poisson's ratio is assumed to be 0.24. The slope layer 2's soil density is assumed to be 2700 Kg/m^3^.

The slope stability analysis problem is regarded as a plain strain problem. The left and right boundaries are restricted horizontally. The bottom boundary is restricted both horizontally and vertically. With the drucker-prager model, as the constitutive model, and with shear strength reduction method based on the finite element analysis, the slope in Figure 14 is analyzed. Assume that the real cohesion and internal friction angle of a slope are *c*_0_ and *φ*_0_, respectively. In the shear strength reduction method, when safety factor is SF, the reduced cohesion and friction angle for analysis are *c*_0_/SF and *φ*_0_/SF.

The Drucker-Prager yield criterion is [24, 25]

(4) $$\begin{array}{c} AI_{1}+\sqrt{J_{2}}-B\leq 0, \end{array}$$

where *I*_1_ = *σ*_1_ + *σ*_2_ + *σ*_3_, *J*_2_ = (1/6)[(*σ*_1_−*σ*_2_)^2^ + (*σ*_2_−*σ*_3_)^2^ + (*σ*_3_−*σ*_1_)^2^].

If we assume that the Drucker-Prager yield surface touches on the interior of the Mohr-Coulomb yield surface, then the expressions [26–28] are

(5) $$\begin{array}{c} A=\frac{2\sin\varphi }{\sqrt{3}\sqrt{3+\sin\varphi }},\;\;B=\frac{6\cdot c\cdot \cos\varphi }{\sqrt{3}\sqrt{3+\sin\varphi }}. \end{array}$$

If the Drucker-Prager yield surface passes through the external apexes of the Mohr-Coulomb yield surface, then [26, 28, 29]

(6) $$\begin{array}{c} A=\frac{2\sin\varphi }{\sqrt{3}\sqrt{3-\sin\varphi }},\;\;B=\frac{6\cdot c\cdot \cos\varphi }{\sqrt{3}\sqrt{3-\sin\varphi }}, \end{array}$$

where *c* is cohesion and *φ* is internal friction angle.

With the drucker-prager model as the constitutive model to analyze the slope under only self-weight in Figure 14, the flow rule which describes the relationship between the plastic potential function and the plastic strain could be found in [23, 30–34]. The incremental elastic-plastic stress-strain relationship and the corresponding elastic-plastic matrix could be found in [23, 35].

**Figure 15:**
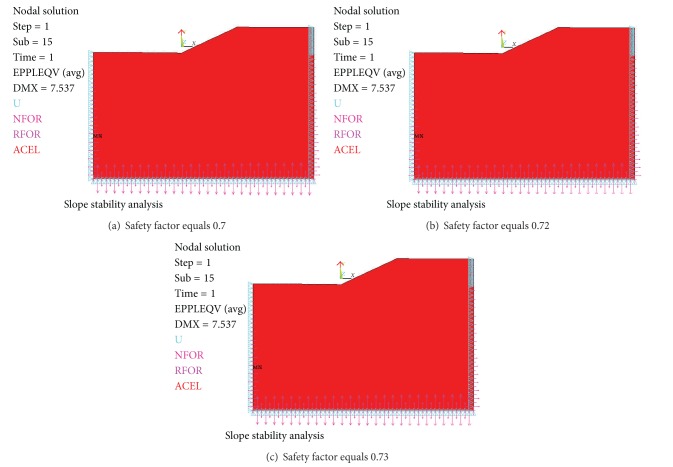
No von Mises plastic strain.

**Figure 16:**
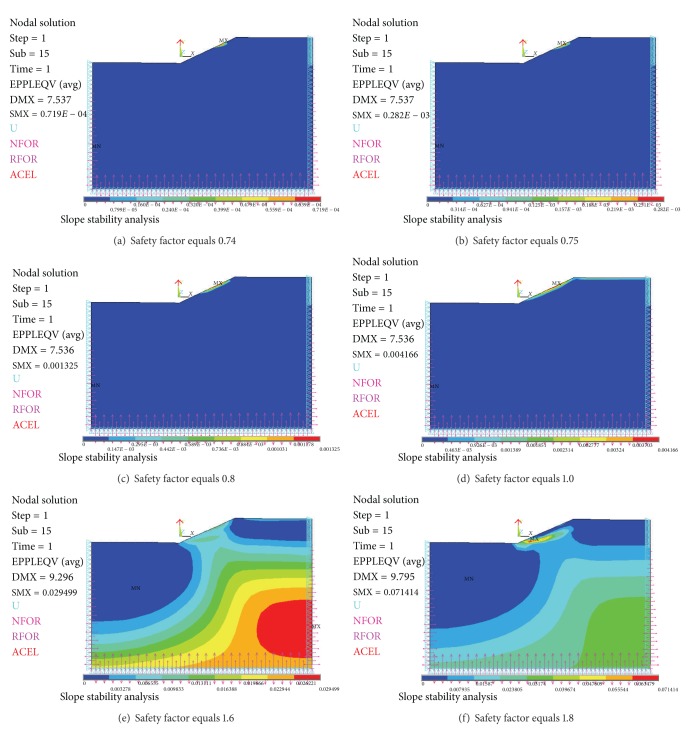
von Mises plastic strain occurs and develops.

**Figure 17:**
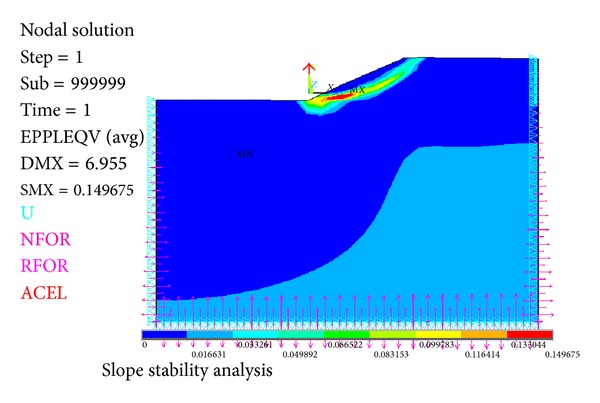
von Mises plastic strain runs through from slope toe to top surface when safety factor equals 2.0.

The results were presented as follows in Figures 15, 16, and 17.

When safety factor is from 0.7 to 0.73, there is no von Mises plastic strain in slope in Figure 15. When safety factor is 0.74, there is local plastic strain occurring in slope in Figure 16. When safety factor is 2.0, von Mises plastic strain runs through from slope toe to top surface in Figure 17.

According to Chen (1975) and Niu (2009), Figure 16 gives lower bound solutions of slope safety factor which are from 0.74 to 1.8. And Figure 17 where von Mises plastic strain runs through from slope toe to top surface gives an upper bound solution of slope safety factor which is 2.0. So the true slope safety factor is likely from 1.8 to 2.0.

### 5.4. Comparisons and Discussions

The obtained minimum safety factor for the above slope stability problem example with Genetic-Traversal Random Search Method is so low when compared with the other methods like slope/w software. This may be due to the fact that the analytical solution is more accurate than Fellenius' slices method. This may also be due to the power of the computer to realize the Genetic-Traversal Random Search Method in the Appendix. The Genetic-Traversal Random Method uses random pick to utilize mutation. Validation of these conclusions will be investigated in the future with more numeric tests.

However the obtained minimum safety factor with Genetic-Traversal Random Search Method is very close to the lower bound solutions of slope safety factor given by the Ansys software.

After computation, there is plastic strain in layer 2 region in some pictures of Figures 16 and 17. This is unreasonable since layer 2 is defined as elastic region in the analysis with Ansys. This phenomenon will be investigated in the future.

## 6. Conclusions

This paper intends to determine a cohesive soil slope safety factor with Fellenius' method, while the 2D critical failure surface is searched with GA. The 2D critical failure surface is represented with real-encoded chromosomes which are potential critical surface locations variables *X*_*c*_ and *X*_*cc*_. GA procedure for searching critical failure surface proceeds with hand calculations. If for future computer automatic search program with GA, program code for inheritance, mutation, selection and crossover, program code for random numbers, and program code for search interval, boundaries will be needed. The minimum safety factor of 1.325 determined by foregoing GA procedure to search the critical slip surface is very close to the minimum safety factor of 1.320 determined by Fellenius' critical slip surface method. After compared with slope/w software, the proposed foregoing GA procedure employed to search the critical failure surface is reasonable, applicable, and effective.

At last, a computer automatic search program (Genetic-Traversal Random Search Method) inspired by GA is made, while in the program random numbers generated by computer and search boundaries are included. The Genetic-Traversal Random Method uses random pick to utilize mutation. In the program, the slope safety factor is given by analytical solution rather than slices method. Results indicate that the new computer automatic search program can give very low safety factor which is about half of the foregoing ones. This may be due to the fact that the analytical solution is more accurate than Fellenius' slices method. This may also be due to the power of the random number generation subprogram, computer operation speed, and Genetic-Traversal Random Method. Further validation of the results will be investigated in the future. However the obtained minimum safety factor with Genetic-Traversal Random Search Method is very close to the lower bound solutions of slope safety factor given by the Ansys software.

## Figures and Tables

**Table 1:** A population of randomly generated individuals.

	*X*_*c*_	*X*_*cc*_	Safety factor
	51	25	2.720523521
	51	41	Safety factor is extremely large, Unreasonable
∗	51	0	1.3317465878
∗	60	−10	1.4074551938
∗	60	25	1.886105777
	60	35	Safety factor is extremely large, Unreasonable
∗	100	−20	2.2757670756
	100	21	2.3067661886
	100	70	Safety factor is extremely large, Unreasonable

**Table 2:** Selected individuals.

Selected individuals		*X*_*c*_	*X*_*cc*_	Safety factor
	∗	51	0	1.3317465878
		60	−10	1.4074551938
		60	25	1.886105777
		100	−20	2.2757670756

**Table 3:** Crossover results.

	*X*_*c*_	*X*_*cc*_	Safety factor
∗	51	−10	1.3799239885
	51	25	2.720523521
	51	−20	1.4447027254
∗	60	0	1.3879596689
	60	−20	1.4333540217
	100	0	2.272574082
	100	−10	2.2729514535
	100	25	2.3308411862

**Table 4:** Mutation results.

	*X*_*c*_	*X*_*cc*_	Safety factor
∗	50	0	1.3425306677
	50	−10	1.4003909843
	51	21	1.7627329758
∗	55	0	1.3332021645
∗	55	−10	1.3615830364
	55	20	1.6493380867

**Table 5:** Selected individuals.

Selected individuals		*X*_*c*_	*X*_*cc*_	Safety factor
	∗	51	0	1.3317465878
		51	−10	1.3799239885
		60	0	1.3879596689
	∗	50	0	1.3425306677
	∗	55	0	1.3332021645
		55	−10	1.3615830364

**Table 6:** Crossover results.

*X*_*c*_	*X*_*cc*_	Safety factor
51	0	1.3317465878
51	−10	1.3799239885
60	0	1.3879596689
50	0	1.3425306677
55	0	1.3332021645
55	−10	1.3615830364

**Table 7:** Mutation results.

	*X*_*c*_	*X*_*cc*_	Safety factor
∗	52	0	1.3264516064
	52	−10	1.3675053085
∗	53	0	1.3254640532
	53	−10	1.3611610322
∗	57	0	1.3503481321
	57	−10	1.3741239435
∗	52	11	1.3501275295
	52	25	2.5159145338
	52	100	Safety factor is extremely large, Unreasonable
	52	15	1.4194510174

**Table 8:** Selected individuals.

Selected individuals		*X*_*c*_	*X*_*cc*_	Safety factor
	∗	51	0	1.3317465878
	∗	50	0	1.3425306677
	∗	55	0	1.3332021645
	∗	52	0	1.3264516064
	∗	53	0	1.3254640532
		57	0	1.3503481321
		52	11	1.3501275295

**Table 9:** Crossover results.

	*X*_*c*_	*X*_*cc*_	Safety factor
	51	11	1.3556896331
	50	11	1.3672906676
	55	11	1.3547892263
∗	53	11	1.3487011763
	57	11	1.3696385778

**Table 10:** Mutation results.

	*X*_*c*_	*X*_*cc*_	Safety factor
	54	21	1.6711004325
∗	54	0	1.3279271582
	54	13	1.3734897577
	54	−10	1.3595160519
	60	−10	1.4074551938

**Table 11:** Selected individuals.

Selected individuals		*X*_*c*_	*X*_*cc*_	Safety factor
		51	0	1.3317465878
		50	0	1.3425306677
		55	0	1.3332021645
		52	0	1.3264516064
	∗	53	0	1.3254640532
		53	11	1.3487011763
		54	0	1.3279271582

**Table 12:** Critical failure surface and minimum safety factor.

	*X*_*c*_	*X*_*cc*_	Safety factor
Completed	53	0	1.3254640532

**Table 13:** Determination of β_1_ and β_2_ with slope angle *α*.

Slope angle *α*	Slope ratio 1 : m	β_1_	β_2_
60°	1 : 0.58	29°	40°
45°	1 : 1.0	28°	37°
33°41′	1 : 1.5	26°	35°
26°34′	1 : 2.0	25°	35°
18°26′	1 : 3.0	26°	35°
14°02′	1 : 4.0	25°	36°
11°19′	1 : 5.0	25°	39°

## Appendix

Safety factor and failure circle determination program developed in Silverfrost FTN95

double precision seed
real nrndl
real N,L,newsafetyfactor
safetyfactor=100000
seed=5.0
gama=20
tanphi=0.5
cohesion=10
h=25
m=2
do 10 I=1,100000
a=0
b=25
c=50
rdn=nrndl(seed)
a=-20∗rdn
rdn=nrndl(seed)
b=25+20∗rdn
rdn=nrndl(seed)
c=50+20∗rdn
if(b==25) b=25.01
x=(c∗c/(2∗b)+(25+b)/2-b/2+a∗a/(50-2∗b))/(c/b+a/(25-b))
y=b/2+(c/b)∗(x-c/2)
r=sqrt((x-c)∗(x-c)+y∗y)
AA=(4∗r∗r-y∗y)∗sqrt(r∗r-y∗y)
BB=(4∗r∗r-(h-y)∗(h-y))∗sqrt(r∗r-(h-y)∗(h-y))
CC=(1/m)∗(2∗r∗r+x∗x)∗sqrt(r∗r-x∗x)
DD=(1/m)∗(2∗r∗r+(m∗h-x)∗(m∗h-x))∗sqrt(r∗r-(m∗h-x)∗(m∗h-x))
EE=y∗asin((sqrt(r∗r-y∗y))/r)
FF=(h-y)∗asin((sqrt(r∗r-(h-y)∗(h-y)))/r)
GG=(x/m)∗asin(x/r)-((m∗h-x)/m)∗asin((m∗h-x)/r)
N=(1/(6∗r))∗(AA+BB+CC-DD)+(r/2)∗(EE-FF+GG)
T=(1/(6∗r))∗(3∗h∗r∗r-y∗y∗y-(h-y)∗(h-y)∗(h-y)-x∗x∗x/m-(m∗h-x)∗(m∗h-x)∗(m∗h-x)/m)
L=r∗(asin((sqrt(r∗r-y∗y))/r)+asin((sqrt(r∗r-(h-y)∗(h-y)))/r))
newsafetyfactor=(gama∗tanphi∗N+cohesion∗L)/
(gama∗T)
if(newsafetyfactor<safetyfactor) safetyfactor=newsafetyfactor
write(∗,∗)safetyfactor
write(∗,∗)a
write(∗,∗)b
write(∗,∗)c
10 continue
end program
real function nrndl(seed)
double precision S,U,V,seed
S=65536.0
U=2053.0
V=13849.0
M=seed/S
seed=seed-M∗S
seed=U∗seed+V
M=seed/S
seed=seed-M∗S
nrndl=seed/S
return
end
